# Method for Improved Performance of Fixed-Gain Self-Alignment in the Temperature Stabilizing State

**DOI:** 10.3390/s20082188

**Published:** 2020-04-13

**Authors:** Inseop Lee, Juhyun Oh, Haesung Yu, Cheonjoong Kim, Sang Jeong Lee

**Affiliations:** 1 Agency for Defense Development, Yuseong P.O. Box 35, Daejeon 34186, Korea; juhyunoh@add.re.kr (J.O.); hae0817@add.re.kr (H.Y.); kcj3651@add.re.kr (C.K.); 2 Chungnam National University, 99 Daehak-ro, Yuseong-gu, Daejeon 34134, Korea; eesjl@cnu.ac.kr

**Keywords:** inertial navigation system, self-alignment, temperature stabilizing error, curve-fitting

## Abstract

Self-alignment (or initial alignment) is the process by which the Inertial Navigation System (INS) is aligned using only measurements from the inertial sensors and the reference navigation information in the stationary state. The main purpose of self-alignment is to calculate the initial attitude of the INS. The accuracy of self-alignment is determined by the performance grade of the inertial sensors, for instance, the accuracy of the horizontal attitude by the horizontal accelerometer and the accuracy of the vertical attitude by the East-axis gyro. Therefore, uncertain errors in the inertial sensors degrade the performance of self-alignment. The focus of this paper is the temperature stabilizing error of accelerometers, a form of uncertain error. An analysis is presented of how the temperature stabilizing error affect the accuracy of self-alignment. From the analysis, a method is proposed to improve performance by curve fitting the horizontal control rates. This is then verified experimentally.

## 1. Introduction

An Inertial Navigation System (INS) provides position, velocity, attitude, angular velocity, and the acceleration of a vehicle by integrating measurements from the system’s Inertial Measurement Unit (IMU), which is essentially a set of accelerometers and gyros. It is widely used in ground, sea, air, and space applications for automated guided vehicle systems [1,2,3,4,5]. In particular, an INS is independent of signals from external sources and is jam-proof, compared to radio navigation systems such as the Global Navigation Satellite System (GNSS). Hence, some vehicle systems that require accurate navigation information employ the INS as their major navigation system.

Unlike a radio navigation system, which calculates its position and velocity via position fixing, the INS relies on dead reckoning and must know its initial condition before starting calculation of the navigation information. This process is called self-alignment, or initial alignment, and is used to obtain the initial attitude of the INS using measurements from the inertial sensors only, in the stationary state. This provides the essential initial condition from which to perform the subsequent recursive calculation of navigation information. The accuracy of self-alignment affects navigation performance; therefore, there has been much research on it. An alignment error estimation method was derived, and an observability analysis was conducted in [6]. Because of unobservable states, a multi-position alignment technique was proposed to enhance the convergence rate [7]. A fast alignment method using disturbance reduction was suggested in [8]. Self-alignment based on low-cost IMU was researched [9]. The forward and backward data processing method was proposed to improve alignment performance [10].

The accuracy of self-alignment is determined by the performance grade of the inertial sensors, for instance, the accuracy of the horizontal attitude by the horizontal accelerometer and the accuracy of the vertical attitude by the East-axis gyro [1,3,4]. In the Gimbaled Inertial Navigation System (GINS), the inertial sensors mounted on the stable platform are always aligned on the navigation frame by adjusting the orientation of the platform and the alignment accuracy is guaranteed. Thus, high-performance horizontal accelerometers and East-axis gyro are implemented in the GINS to improve alignment performance. In the Strap-down Inertial Navigation System (SINS), the measurements from the inertial sensors mounted directly on the vehicle body may be resolved into the navigation frame using the current body attitude with respect to this frame. The calculated accelerations are influenced by the measurements from every 3-axis sensor; therefore, the choice of inertial sensors for strap-down mechanization is dependent on the application.

The attitude matrix that defines the relationship between the body frame and the navigation frame is determined by measurements from the gyros, and then the velocity can be calculated by performing integration on the acceleration transformed into the navigation frame using this matrix. Because the true components of the gravity in the north and east directions are nominally zero, any departure from zero in the accelerometer measurements resolved in these directions may be interpreted as an error. The self-alignment of the SINS calls for the design of a state feedback control so that the error becomes zero and the attitude matrix is obtained accurately.

For the process of self-alignment, a control loop is designed with the assumption that the output of the accelerometers is the component of gravity that includes random constant bias. However, the actual output of the accelerometer varies due to changes in internal and external temperatures while the temperature is stabilizing. This change in temperature means that the accuracy needed in self-alignment cannot be satisfied by the method of the existing design.

To mitigate the temperature drift error of the accelerometer, research on compensation methods has been conducted. A direct method is to add a temperature control system [11]. Considering hardware feasibility, the compensation method using a mathematical model is commonly used in the accelerometer design [12,13]. Once accelerometers are assembled to an INS, this compensation method is not sufficient because the temperature environment is changed. Moreover, the alignment process is generally performed right after the INS turn on, then the temperature stabilizing error significantly affects the alignment accuracy. However, research on the self-alignment technique with consideration of the temperature drift error has not yet been published.

This paper focuses on the self-alignment technique with the assumption that accelerometers are provided by a manufacturer. First, we analyze the bias error of an accelerometer in the temperature stabilizing state in an experiment and introduce a bias-error model based on the analysis. Regarding the error model, we present an analysis of how errors in the temperature stabilizing state of the accelerometer affect the accuracy of self-alignment theoretically and in a simulation. Subsequently, we propose an improved method for the estimation of heading and present the analysis and experimental results.

In Section 2 of this paper, the experimental results and bias error model of an accelerometer in the temperature stabilizing state are shown. Section 3 presents a theoretical analysis of how the bias error affects the self-alignment performance using the fixed-gain alignment method, and the analysis results are supported by the simulation results. In Section 4, the improved self-alignment method is proposed after the conventional method is explained. Section 5 provides the experimental results for the improved method, and the conclusions follow in Section 6.

## 2. Temperature Stabilizing Error of an Accelerometer and its Error Model

**Figure 1:**
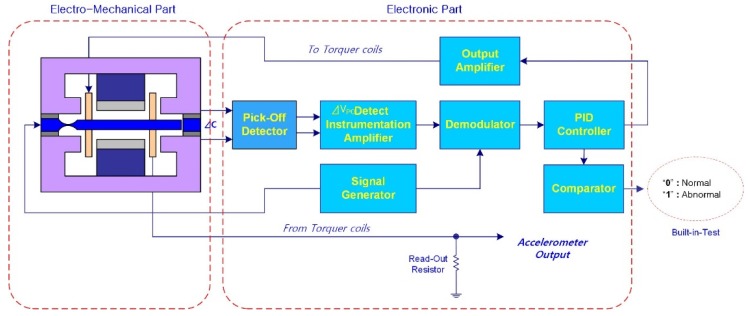
Schematic diagram of an accelerometer structure. An acceleration occurs, the pendulum tries to move in the opposite direction. Electronic rebalance loop makes the pendulum maintain to zero point. Acceleration is measured by the feedback signal. The variation of pendulum characteristics caused by temperature change results in temperature stabilizing error.

An accelerometer measures acceleration using the principle that the force applied to a mass is proportional to its acceleration in the same direction. An accelerometer consists of electro-mechanical and electronic parts, as depicted in Figure 1. The electro-mechanical part contains sensors that detect acceleration and a torquer coil to restore a pendulum to the zero point. The electronic part is a servo feedback loop used to process the acceleration signal. As shown in Figure 1, the pendulum moves as acceleration occurs in the +g direction and the spatial displacement of the pendulum in the capacitive system produces a change in an electrical charge. This change is sensed by the pick-off and transferred to the electronic part. The electronic rebalance loop feeds the signal back to the torquer coil. This signal generates the electromagnetic force needed to maintain the pendulum at the zero point. The feedback signal is proportional to the acceleration applied and it may be measured from the read-out resistor. Thus, the signal processing unit is also required to measure an accurate acceleration signal in the INS.

Based on the mechanism above, the general error model of the accelerometer is formulated as follows.

(1) $$\delta a^{b}=\delta b+S\times f^{b}+C\times f^{b}+A\times f_{i}\times f_{j}$$

where $\delta a^{b}$ means accelerometer error on the body frame and $f_{i},$ $f_{j}$ means acceleration on the i, j axis. Here, δb denotes the bias error, and S, C, and A represent the scale factor error, cross-coupling error, and vibro-pendulous error, respectively. Each error factor is modeled as a random constant. Because self-alignment performance is the focus in this paper, the scale factor error, cross-coupling error, and vibro-pendulous error were neglected and the error model simplified as follows.

(2) $$\delta a^{b}=\delta b$$

**Figure 2:**
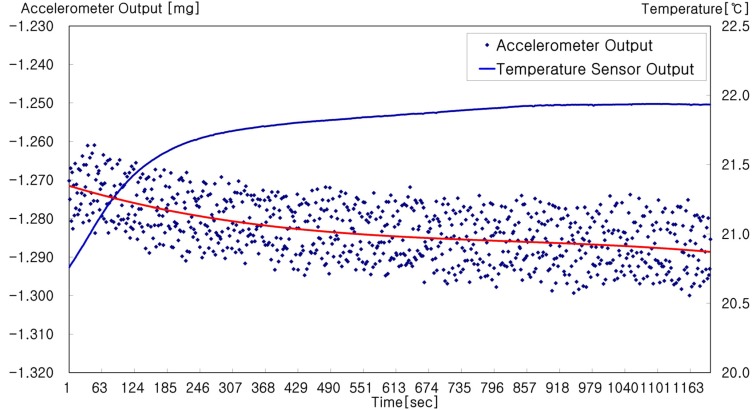
Accelerometer output in the temperature stabilizing state. Right after an accelerometer is powered on, the temperature inside increases rapidly. That temperature change causes an accelerometer temperature stabilizing error.

Bias error is caused by the difference from the zero point of the pendulum and the pick-off signal. Temperature change causes a variation of the pendulum stress and the pick-off sensitivity, and a control signal is generated to adjust the zero point of the pendulum. This becomes the bias error of the accelerometer in the stationary state. Therefore, an accelerometer needs to be designed, considering both the electro-mechanical and electronic parts simultaneously, to reduce the bias error. In particular, the characteristics of both parts should be constant so that the INS maintains the same performance over its full range of operating temperatures. However, it is impossible to design such an accelerometer and it is generally considered to model the bias error as a function of temperature. This compensation method may not be applied while the temperature of the accelerometer is stabilizing because the error is modeled after temperature stabilization is achieved. When the accelerometer is powered on, the temperature of the electronic part increases rapidly and unmodeled bias error is generated in this state. Figure 2 shows the experimental result of accelerometer measurements made during the temperature stabilizing state. The measurements converge to the bounded value over time. Thus, it is not reasonable to model the bias error as a random constant.

In Figure 2, the left and right axes represent the output and temperature, respectively, of the accelerometer. The small dots are the experimental results of the accelerometer measurements and the red solid line starting from −1.272 is their average. Consequently, it is shown that the measurements converge around −1.282. Most accelerometers exhibit dynamics similar to that above, even though the initial amplitude and the convergence behaviors are different. Moreover, the accelerometer output shown in Figure 2 is of measurements compensated using a temperature function that is practical for use in self-alignment. The bias error in the temperature stabilizing state can be modeled as follows.

(3) $$\delta a^{n}=\left(1-e^{-\frac{t}{T_{i}}}\right)\delta a_{v}$$

where $\delta a^{n}$ denotes the accelerometer error on the navigation frame. $\delta a_{v}$ is the temperature stabilizing bias (final convergence value) and $T_{i}$ is the time constant. Each has a different value according to the accelerometer. It is known that QA-3000, a small-size high-grade accelerometer, also has temperature stabilizing bias error and compensate the error through modeling like Equation (3) [14,15].

## 3. Performance Analysis of Self-Alignment

### 3.1. Control Loop of Self-Alignment

Two methods are used to align the INS: fixed-gain and variable gain. In this paper, a second-order fixed-gain controller was adopted to analyze the self-alignment performance during the temperature stabilizing state.

**Figure 3:**
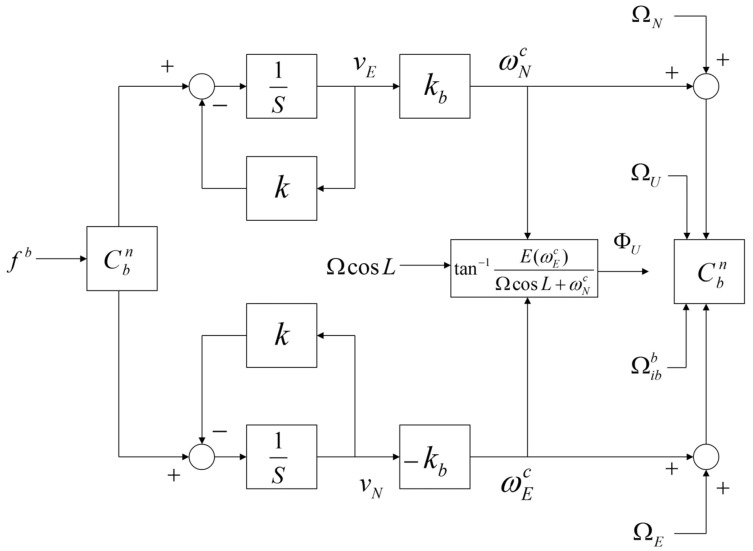
Block diagram of the self-alignment loop. Control angular rates ($\omega_{N}^{c}$, $\omega_{E}^{c}$) are calculated by horizontal velocities ($v_{E}$, $v_{N}$) to make these velocities zero. A horizontal attitude (roll or pitch) is calculated by either control rate. Vertical attitude (heading) is calculated by both control rates.

Figure 3 shows a block diagram of the self-alignment loop using the second-order fixed-gain controller. The controller provides the control signals $\omega_{E}^{c}$ and $\omega_{N}^{c}$, which are used to make the measurement of the horizontal accelerometer $f^{n}$ zero in the navigation frame. The performance of the horizontal axis alignment (roll and pitch) is based on $C_{b}^{n}$, the transformation matrix between the body and navigation frames, which is calculated using the gyro output $\Omega_{ib}^{b}$ and the control signals. The vertical axis attitude (heading) $\Phi_{U}$ is calculated using the control signal as described in Figure 3. In Figure 3, k, and $k_{b}$ are the alignment-loop coefficients, $v_{E}$ and $v_{N}$ are the velocities in the navigation frame, Ω is the Earth’s rotation rate, and L is the latitude. In addition, $\Omega_{E}$, $\Omega_{N}$, and $\Omega_{U}$ denote the angular velocities in the navigation frame and $\Omega_{ib}^{b}$ is the angular velocity of the gyro.

**Figure 4:**
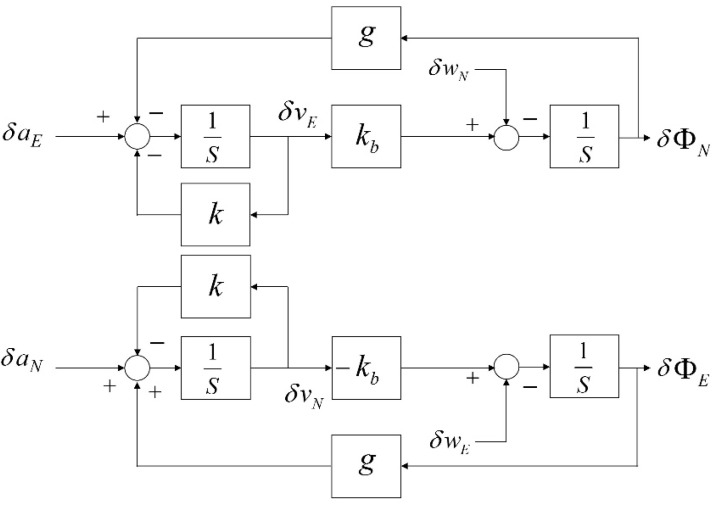
Error model of the self-alignment loop. While E(N)-axis velocity error $\delta v_{E}\left(\delta v_{N}\right)$ induces N(E)-axis attitude error ${\delta \Phi }_{N}$(${\delta \Phi }_{E}$), vertical attitude is not related to horizontal velocity errors.

Figure 4 shows a block diagram of the error model of the self-alignment loop. The error equations are expressed as follows in the Laplace transform.

(4) $$\delta v_{E}\left(s\right)=\frac{s\delta a_{E}\left(s\right)+g\delta \omega_{N}\left(s\right)}{s^{2}+ks+k_{b}g}$$

(5) $$\delta v_{N}\left(s\right)=\frac{s\delta a_{N}\left(s\right)-g\delta \omega_{E}\left(s\right)}{s^{2}+ks+k_{b}g}$$

(6) $${\delta \Phi }_{E}\left(s\right)=\frac{-k_{b}\delta a_{N}\left(s\right)-\left(s+k\right)\delta \omega_{E}\left(s\right)}{s^{2}+ks+k_{b}g}$$

(7) $${\delta \Phi }_{N}\left(s\right)=\frac{k_{b}\delta a_{E}\left(s\right)-\left(s+k\right)\delta \omega_{N}\left(s\right)}{s^{2}+ks+k_{b}g}$$

where g is the gravitational acceleration, $\delta a_{E}$ and $\delta a_{N}$ are the acceleration error, $\delta \omega_{E}$ and $\delta \omega_{N}$ are the gyro error, $\delta v_{E}$ and $\delta v_{N}$ are the velocity error, and ${\delta \Phi }_{E}$ and ${\delta \Phi }_{N}$ are the attitude error. These equations show that the controller adopted a second-order low-pass filter and that the cutoff frequency $\omega_{c}=\sqrt{k_{b}g}$, can be determined by the value $k_{b}$, which is set for the self-alignment loop. Thus, $\omega_{c}$ should be selected to eliminate the sinusoid of the accelerometer and gyro measurement. Because $\omega_{c}$ affects the convergence behavior, the alignment loop may be disturbed by noise if the value of $\omega_{c}$ is too small. Therefore, it is necessary to select $\omega_{c}$ optimally, considering the filtering performance and the response characteristics of the alignment loop.

### 3.2. Performance Degradation due to Temperature Stabilizing Error

The error model of the accelerometer and gyro was added to Equations (4) and (5). For the performance analysis of the accelerometer-bias error in the temperature stabilizing state, the accelerometer error is modeled as in Equation (3) and the gyro error is assumed to be a random constant. The error models of the accelerometer and gyro can be written in terms of Laplace transforms, as follows.

(8) $$\delta a^{n}=\frac{\frac{1}{T_{i}}}{s\left(s+\frac{1}{T_{i}}\right)}\delta a_{v}$$

(9) $$\delta \omega^{n}=\frac{1}{s}\delta \omega_{v}$$

By applying the above model, we obtain the following equations. In Equations (10)–(13), $\delta a_{iv}$ and $\delta \omega_{iv}$ are the accelerometer and gyro bias, respectively, on the i-axis.

(10) $$\delta v_{E}\left(s\right)=\frac{\frac{\delta a_{Ev}}{T_{i}}}{\frac{1}{T_{i}^{2}}-\frac{k}{T_{i}}+k_{b}g}\left[\frac{1}{s+\frac{1}{T_{i}}}-\frac{s+\left(k-\frac{1}{T_{i}}\right)}{s^{2}+ks+k_{b}g}\right]+\frac{\delta \omega_{Nv}}{k_{b}}\left[\frac{1}{s}-\frac{s+k}{s^{2}+ks+k_{b}g}\right]$$

(11) $$\delta v_{N}\left(s\right)=\frac{\frac{\delta a_{Nv}}{T_{i}}}{\frac{1}{T_{i}^{2}}-\frac{k}{T_{i}}+k_{b}g}\left[\frac{1}{s+\frac{1}{T_{i}}}-\frac{s+\left(k-\frac{1}{T_{i}}\right)}{s^{2}+ks+k_{b}g}\right]-\frac{\delta \omega_{Ev}}{k_{b}}\left[\frac{1}{s}-\frac{s+k}{s^{2}+ks+k_{b}g}\right]$$

(12) $${\delta \Phi }_{E}\left(s\right)=-k_{b}\delta a_{Nv}[\frac{1}{k_{b}g}\frac{1}{s}-\frac{1}{\frac{1}{T_{i}{}^{2}}-\frac{k}{T_{i}}+k_{b}g}\left(\frac{1}{s+\frac{1}{T_{i}}}-\frac{\left(\frac{\frac{1}{T_{i}}\left(k-\frac{1}{T_{i}}\right)}{k_{b}g}\right)s+\left(\frac{\frac{1}{T_{i}}\left(k^{2}-\frac{k}{T_{i}}-k_{b}g\right)}{k_{b}g}\right)}{s^{2}+ks+k_{b}g}\right)]-\frac{\delta \omega_{Ev}}{k_{b}g}\left[\frac{k}{s}-\frac{ks+\left(k^{2}-k_{b}g\right)}{s^{2}+ks+k_{b}g}\right]$$

(13) $${\delta \Phi }_{N}\left(s\right)=k_{b}\delta a_{Ev}[\frac{1}{k_{b}g}\frac{1}{s}-\frac{1}{\frac{1}{T_{i}{}^{2}}-\frac{k}{T_{i}}+k_{b}g}\left(\frac{1}{s+\frac{1}{T_{i}}}-\frac{\left(\frac{\frac{1}{T_{i}}\left(k-\frac{1}{T_{i}}\right)}{k_{b}g}\right)s+\left(\frac{\frac{1}{T_{i}}\left(k^{2}-\frac{k}{T_{i}}-k_{b}g\right)}{k_{b}g}\right)}{s^{2}+ks+k_{b}g}\right)]-\frac{\delta \omega_{Nv}}{k_{b}g}\left[\frac{k}{s}-\frac{ks+\left(k^{2}-k_{b}g\right)}{s^{2}+ks+k_{b}g}\right]$$

In the second-order characteristic equation, the real part of roots is negative, which is because the alignment loop gain k and $k_{b}$ are always positive, and $k^{2}-4k_{b}g$ is generally negative, for example, k is 3.5 and $k_{b}$ is 0.8 in the alignment loop design. Consequently, the alignment loop converges rapidly when the time constant of the accelerometer temperature stabilizing bias is larger than the time constant of the characteristic equation. The fine alignment needed to calculate the vertical attitude is performed after stabilizing the horizontal attitude through a coarse alignment of about 30 sec. Hence, it seems reasonable to analyze how the errors that occur during the temperature stabilizing state of the accelerometers affect the accuracy of self-alignment without the second-order terms in Equations (10)–(13). Equations (10) to (13) can be written in terms of an Inverse Laplace transform, as follows.

(14) $$\delta v_{E}\left(t\right)=\frac{\frac{\delta a_{Ev}}{T_{i}}}{\frac{1}{T_{i}^{2}}-\frac{k}{T_{i}}+k_{b}g}e^{-\frac{t}{T_{i}}}+\frac{\delta \omega_{Nv}}{k_{b}}$$

(15) $$\delta v_{N}\left(t\right)=\frac{\frac{\delta a_{Nv}}{T_{i}}}{\frac{1}{T_{i}^{2}}-\frac{k}{T_{i}}+k_{b}g}e^{-\frac{t}{T_{i}}}-\frac{\delta \omega_{Ev}}{k_{b}}$$

(16) $${\delta \Phi }_{E}\left(t\right)=-k_{b}\delta a_{Nv}\left[\frac{1}{k_{b}g}-\frac{1}{\frac{1}{T_{i}^{2}}-\frac{k}{T_{i}}+k_{b}g}e^{-\frac{t}{T_{i}}}\right]-\frac{\delta \omega_{Ev}k}{k_{b}g}$$

(17) $${\delta \Phi }_{N}\left(t\right)=k_{b}\delta a_{Ev}\left[\frac{1}{k_{b}g}-\frac{1}{\frac{1}{T_{i}^{2}}-\frac{k}{T_{i}}+k_{b}g}e^{-\frac{t}{T_{i}}}\right]-\frac{\delta \omega_{Nv}k}{k_{b}g}$$

Equations (14) to (17) shows that the velocity and attitude errors converge over time due to the time constant of the accelerometer bias error in the temperature stabilizing state and that the amplitude is determined by the alignment loop coefficients and a time constant of the accelerometer bias error in Equation (3). The velocity and attitude errors on the horizontal axis, are barely affected because the amplitude $\delta a^{v}$ is not large compared to the other errors. However, the vertical attitude is determined using the horizontal axis control signal and can be greatly affected by even a small variation of the velocity. The error of the vertical attitude, which is derived by applying the perturbation method to $\Phi_{U}$, is as follows.

(18) $$\delta \Phi_{U}=\frac{\delta \omega_{E}^{c}}{\Omega cosL}$$

It can be rewritten by introducing the North-axis velocity error as follows.

(19) $$\delta \Phi_{U}=\frac{-\frac{\frac{k_{b}\delta a_{Nv}}{T_{i}}}{\frac{1}{T_{i}^{2}}-\frac{k}{T_{i}}+k_{b}g}e^{-\frac{t}{T_{i}}}+\delta \omega_{Ev}}{\Omega cosL}$$

The above equation represents the relationship between the accelerometer bias error in the temperature stabilizing state and the vertical axis attitude error. This shows that a small accelerometer bias error in the temperature stabilizing state can yield a large vertical axis attitude error. It also shows that the amplitude of an additional vertical axis attitude error, that is affected by the accelerometer bias error, is determined by the amplitude and the time constant in Equation (3).

### 3.3. Simulation of the Attitude Error Induced by the Temperature Stabilizing Error

A simulation regarding the relationship between the accelerometer bias error in the temperature stabilizing state and the attitude error was performed. We assumed that the self-alignment loop in Figure 3 had a corner frequency of 2 Hz and that the accelerometer bias error in the temperature stabilizing state had a time constant of 300 s and an amplitude of 30 μg for the simulation. In addition, we assumed that there were no other errors except the horizontal accelerometer bias error and that true roll, pitch, and heading angles of the INS at self-alignment were zero. The execution cycle of the self-alignment loop was 200 Hz.

**Figure 5:**
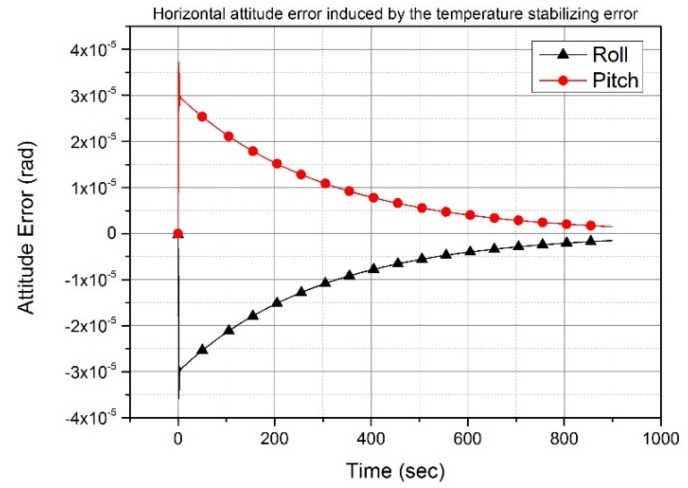
Horizontal attitude error induced by the temperature stabilizing error. Roll and pitch errors decrease exponentially.

Figure 5 shows the roll and pitch angles with accelerometer bias error in the temperature stabilizing state. The roll angle has the initial angle of minus 30 μrad and gradually decreases to zero. The pitch angle has the same convergence behavior in amplitude as the roll angle, even though the sign is different. This simulation result can be obtained through a calculation using Equations (20) and (21). Equations (20) and (21) are easily derived from Equations (16) and (17) if the terms related to the time constant in Equations (16) and (17) is ignored. This is because the corner frequency is very large compared to the other terms. Equations (20) and (21) shows the approximate convergence behavior of the roll and pitch angles.

(20) $$\delta \Phi_{E}\approx -\frac{\delta a_{Nv}}{g}\left[1-e^{-\frac{t}{T_{i}}}\right]$$

(21) $$\delta \Phi_{N}\approx \frac{\delta a_{Ev}}{g}\left[1-e^{-\frac{t}{T_{i}}}\right]$$

(22) $$\delta \Phi_{U}\approx -\frac{\delta a_{Nv}}{T_{i}g\Omega cosL}e^{-\frac{t}{T_{i}}}$$

**Figure 6:**
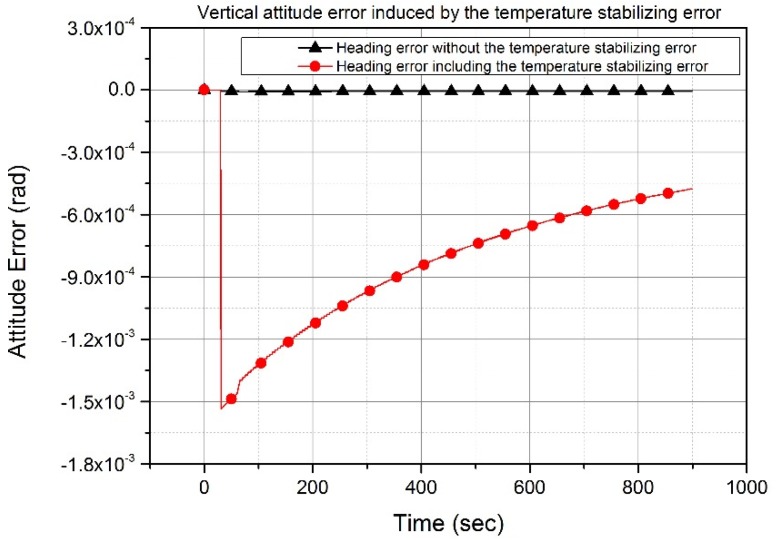
Heading errors depending on the temperature stabilizing error. Temperature stabilizing error induces exponentially decreasing heading error, which is greater than the horizontal attitude error in Figure 5.

In Figure 6, the black line (triangle symbol) indicates the heading error and the red line (circle symbol) indicates the heading error with the accelerometer bias error in the latitude of 36°. The heading angle calculated with Equation (19) and using a time constant and the amplitude of the accelerometer bias is 1.5 mrad at 30 sec and 1.4 mrad at 60 sec, respectively. This calculated heading angle is the same as that obtained through the simulation illustrated in Figure 6. The heading angle decreases with time because it is calculated by averaging the control rate, as shown in Figure 3. The simulation results in Figure 5 and Figure 6 show that the equations representing the relationship between the accelerometer bias error in the temperature stabilizing state and the attitude error are correct.

## 4. Improved Method for Self-Alignment

### 4.1. Conventional Total Average Method for Heading Estimation

So far, a gyro random walk was excluded from the gyro error model. However, gyro random walks do exist in the real world, and we have to take into account that error. Considering these random walks, the instant measurement of the horizontal control rate degrades the alignment accuracy because the variation of the horizontal control rates could be quite large. Therefore, a total average is generally applied to the horizontal control rate to prevent degrading the alignment accuracy (see Equations (23) and (24)).

(23) $$\hat{\beta_{E}}=\frac{1}{T}\int_{0}^{T}\hat{\omega_{E}^{c}}\left(t\right)dt$$

(24) $$\hat{\beta_{N}}=\Omega_{N}+\frac{1}{T}\int_{0}^{T}\hat{\omega_{N}^{c}}\left(t\right)dt$$

where T is the total averaging time. Finally, a heading angle is estimated using Equation (25).

(25) $$\Phi_{U}=\arctan\left(\frac{\hat{\beta_{E}}}{\hat{\beta_{N}}}\right)$$

The heading error induced by the temperature stabilizing error and gyro error diminishes as the averaging time goes on due to the smoothing effect. Thus, self-alignment should be performed for a long time, considering the presence of temperature stabilizing errors and gyro random walks. The heading error can be expressed as in Equation (26).

(26) $$\delta \Phi_{U}=\frac{\frac{\frac{k_{b}\delta a_{Nv}}{T_{i}}}{\frac{1}{T_{i}^{2}}-\frac{k}{T_{i}}+k_{b}g}\left(e^{-\frac{T}{T_{i}}}-1\right)}{\Omega cosL}+\frac{\delta \omega_{Erw}}{\sqrt{T}\Omega cosL}+\frac{\delta \omega_{Eb}}{\Omega cosL}$$

where $\delta \omega_{Erw}$ is gyro random walk error and $\delta \omega_{Eb}$ is gyro bias. Note that, $\delta \omega_{Erw}$ and $\delta \omega_{Eb}$ come from $\delta \omega_{Ev}$. Equation (26) shows that the influence of the temperature stabilizing error, and a gyro random walk on the heading error diminishes with an increase in the alignment time T and that the heading error is eventually determined by the gyro bias. To guarantee accurate heading and to reduce the alignment time, it is necessary that the gyro random walks and the temperature stabilizing error of accelerometers are improved. Because gyro random walks are dependent on the physical characteristics of a gyroscope, it is impossible to improve gyro random walk conditions through a self-alignment technique. Therefore, we present a new technique to prevent the degradation of the alignment accuracy and to reduce alignment time, under the assumption that the influence of gyro random walks on the heading error is quite small compared to the temperature stabilizing error.

### 4.2. Curve-Fitting Method for Heading Estimation

**Figure 7:**
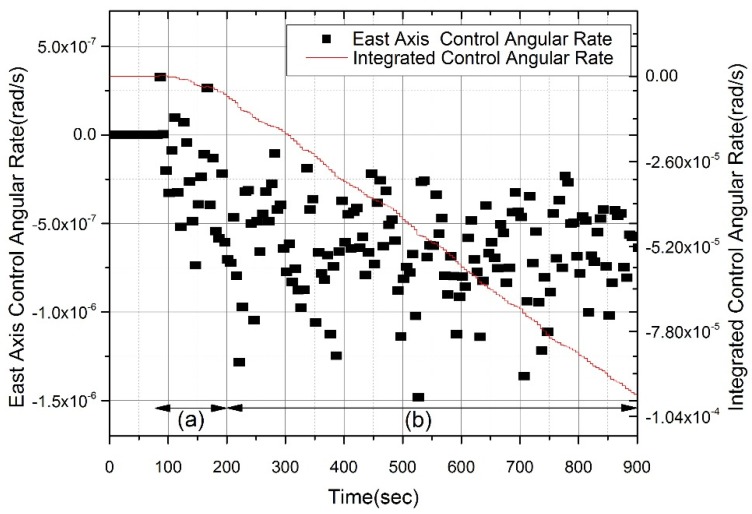
Control angular rate and its integration during temperature stabilization. The control rate changes exponentially in (a) region because of the temperature stabilizing error. The control rate is stabilized in (b) region and its integrated curve is almost linear. The control rate is overall scattered by gyro random walks, and its integrated curve has a minor deviation in both (a) and (b) region.

Here, we propose a method for the curve fitting of the horizontal control rate in the temperature stabilizing state of the accelerometer. Figure 7 shows the East-axis control rate (black dots that are scattered by gyro random walks), and its integrated result (red line). The suggested method involves integrating the horizontal control rate, fitting the curve on the integrated line, and then using the slope of the fitted curve to estimate the heading angle. Because self-alignment is performed while the vehicle is stationary, the true value of the horizontal control rate should be constant, and its integrated result should also be a linear function. This method will improve alignment performance compared to total-averaging, due to a smoothing effect.

Equation (27) is the integrated result of the horizontal control rate, $\hat{\omega_{E}^{c}}$. This equation includes four terms. The first is a linearly increasing term that includes the true value of $\hat{\omega_{E}^{c}}$ and the gyro bias. The second term is a constant. The third is a decay term generated by the temperature stabilization of the accelerometers. The fourth term is generated by a gyro random walk. Applying Taylor series expansion to the exponential function, Equation (27) can be expressed as Equation (28). After total-averaging using Equation (23), the control rate is expressed as Equation (29). The conventional method uses Equation (29) to obtain the horizontal control rate. Therefore, heading accuracy could be degraded and the alignment time could be made longer by the temperature stabilizing error, which is the second term of Equation (29).

(27) $$\hat{\alpha_{E}}\left(t\right)=\int_{0}^{t}\hat{\omega_{E}^{c}}\left(\tau \right)d\tau \;=\left(\omega_{E}^{c}+\delta \omega_{Eb}\right)t-\frac{k_{b}\delta a_{Nv}}{\frac{1}{T_{i}^{2}}-\frac{k}{T_{i}}+k_{b}g}+\frac{k_{b}\delta a_{Nv}}{\frac{1}{T_{i}^{2}}-\frac{k}{T_{i}}+k_{b}g}e^{-\frac{t}{T_{i}}}+\int_{0}^{t}\delta \omega_{Erw}\left(\tau \right)d\tau$$

(28) $$\hat{\alpha_{E}}\left(t\right)=\left(\omega_{E}^{c}+\delta \omega_{Eb}-\frac{1}{T_{i}}\right)t+A\left(\frac{t^{2}}{2!T_{i}^{2}}-\frac{t^{3}}{3!T_{i}^{3}}+\cdots \right)+\int_{0}^{t}\delta \omega_{Erw}\left(\tau \right)d\tau$$

(29) $$\hat{\beta_{E}}=\left(\omega_{E}^{c}+\delta \omega_{Eb}-\frac{1}{T_{i}}\right)+A\left(\frac{T}{2!T_{i}^{2}}-\frac{T^{2}}{3!T_{i}^{3}}+\cdots \right)+\frac{1}{T}\int_{0}^{T}\delta \omega_{Erw}\left(t\right)dt$$

where $A=\frac{k_{b}\delta a_{Nv}}{\frac{1}{T_{i}^{2}}-\frac{k}{T_{i}}+k_{b}g}$ in Equations (28) and (29).

To improve self-alignment performance, we suggest using the curve fitting method, which includes the construction of a measurement equation and estimation of a first-order coefficient using the Least Squares Method. The measurement equation z=Hx+ε, used for curve fitting can be expressed as Equation (30). In Equation (30), the measurement vector component $\hat{\alpha_{E}}\left(t_{i}\right)$ refers to the integrated value of the horizontal control rate at $t_{i}$, and ε indicates the error term. H is a measurement model for linear function curve-fitting, ‘a’ is the slope, and ‘b’ is the y-intercept of the fitted-curve. Applying the Least Squares Method to Equation (30), a state-space equation can be expressed as in Equation (31). Finally, the control rates of the horizontal axes (East and North) are estimated using Equation (31). Using Equation (31), the estimate of the control rate is ‘a’ and the higher-order terms generated by the temperature stabilizing error are estimated as the error term ε.

(30) $$\left[\begin{array}{c} \begin{array}{c} \hat{\alpha_{E}}\left(t_{0}\right)\\ \hat{\alpha_{E}}\left(t_{1}\right) \end{array}\\ .\\ .\\ .\\ \hat{\alpha_{E}}\left(t_{n}\right) \end{array}\right]=\left[\begin{array}{c} \begin{array}{c} \begin{array}{cc} t_{0} & 1 \end{array}\\ \begin{array}{cc} t_{1} & 1 \end{array} \end{array}\\ \begin{array}{cc} . & . \end{array}\\ \begin{array}{cc} . & . \end{array}\\ \begin{array}{cc} . & . \end{array}\\ \begin{array}{cc} t_{n} & 1 \end{array} \end{array}\right]\left[\begin{array}{c} a\\ b \end{array}\right]+\left[\begin{array}{c} \epsilon_{0}\\ \epsilon_{1}\\ .\\ .\\ .\\ \epsilon_{n} \end{array}\right]$$

(31) $$\hat{x}=\left[\begin{array}{c} a\\ b \end{array}\right]={\left(H^{T}H\right)}^{-1}H^{T}z$$

## 5. Experimental Results

**Figure 8:**
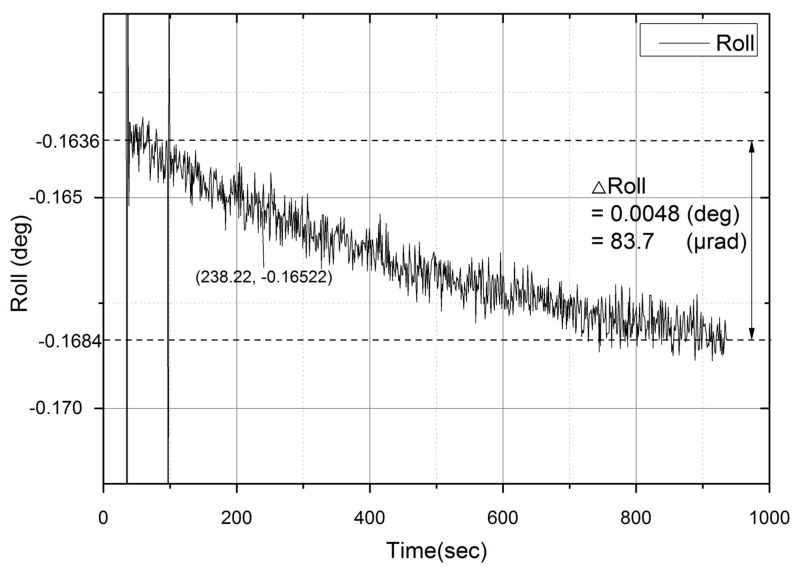
The drift of a roll angle during a self-alignment experiment. Even though the Inertial Navigation System (INS) was stationary, the roll angle gradually decreased, and the amount of the decrement became 0.0048° at 900 sec. This roll angle variation is caused by accelerometer temperature stabilizing error.

To verify the improvement of the fixed-gain self-alignment using the curve fitting method, an INS experiment was performed. The attitude of the INS was fixed at around [0°, 0°, 90°] on an East North Up (ENU) reference frame. The experimental sequence was: power-on the INS, perform self-alignment for 15 min, reboot, and repeat these steps 38 times. The true value of the heading was estimated by averaging the 38 results. Because the temperature stabilizing error of the X-axis (pointing to N-axis) accelerometer affects the Y-axis (pointing to E-axis) gyro error, when the ENU attitude was 0°, 0°, and 90°, the roll angle variation had to be observed to verify how the temperature stabilizing error affected the heading error. Figure 8 shows the roll angle variation in the first run of the experiment. After aligning the horizontal axes, the X-axis acceleration was represented as below:

(32) $$f_{xb}=-g\Phi_{y}\left(X\right)$$

where $f_{xb}$ is the X-axis acceleration on the body frame, g is the gravitational acceleration, and $\Phi_{y}$ is the Y-axis attitude (i.e., roll angle). From Equation (32) and Figure 8, the temperature stabilizing error appears to be about 83.7 μg.

**Figure 9:**
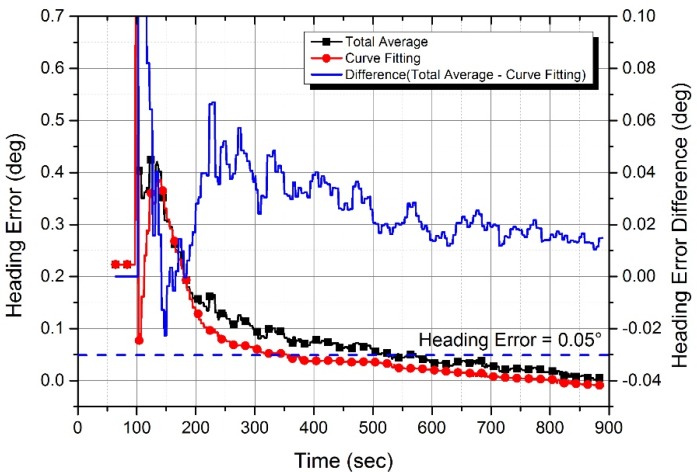
Heading error estimate from the INS experiment results. The red line converges to almost 0° smoothly, while the black line has local fluctuation, which is caused by a gyro random walk. The longer the total average time is, the less the fluctuation becomes. After 180 sec, the heading error of the curve fitting method is less than that of the total average method. The time needed for the heading error to reach within 0.05° is 355 sec for the curve fitting method, and 529 sec for the total average method.

In the experiment, while the self-alignment was performed 38 times sequentially, temperature stabilizing occurred. This means the temperature stabilizing error reveals itself most significantly in the first run. The first alignment result is displayed in Figure 9. The heading error of the curve fitting method (red line, circle symbol) converges to almost 0°, which is smooth compared to that of the total average method (black line, square symbol). The black line has local fluctuation, which is caused by a gyro random walk. The longer the total average time is, the less the fluctuation becomes.

The blue line is the difference between the two heading errors. The blue line over 0 means that the curve fitting method is better than the total average method. In 140–180 sec, the blue line is under 0 because of the curve fitting error. This is because the integration time of the control rate (see Figure 7) is too short to estimate control rate. After 180 sec, the blue line is positively decreased. Note that the amplitude of fluctuation gets reduced as the total average time is longer. From a time perspective, the time needed for the heading error to reach within 0.05° is reduced to 174 sec (355 sec for the curve fitting and 529 sec for the total average). Therefore, in the presence of the temperature stabilizing error, the heading estimation is improved and the alignment time is reduced for the curve fitting method, compared to using a conventional total average method.

## 6. Discussion

In this paper, the bias error of accelerometers in the temperature stabilizing state was analyzed experimentally, and an error model was introduced. It is shown that the performance of self-alignment is degraded when temperature stabilizing errors exist. We proposed the use of a curve-fitting method for the estimation of heading to improve the performance of fixed-gain self-alignment during the temperature stabilizing state of an accelerometer. The experimental results presented show that heading estimation is improved and that the alignment time is reduced by curve-fitting compared to the use of the conventional total average.
